# Assessing the impact of a negative air ionization system on particulate matter and gaseous pollutants in the swine farrowing environment

**DOI:** 10.1371/journal.pone.0316914

**Published:** 2025-02-24

**Authors:** Magnus R. Campler, Yi-Fan Shen, Leonardo M. Klüppel, Andréia G. Arruda

**Affiliations:** 1 Department of Veterinary Preventive Medicine, The College of Veterinary Medicine, The Ohio State University, Columbus, Ohio, United States of America; 2 Department of Management and Human Resources, Fisher College of Business, The Ohio State University, Columbus, Ohio, United States of America; CREA: Consiglio per la ricerca in agricoltura e l'analisi dell'economia agraria, ITALY

## Abstract

Air quality on swine farms has long been a concern for both human and swine health as it has been previously linked with respiratory issues; the main cause being the inhalation of small airborne particulate matter (PM) < 10 μm in diameter. Negative ionizing systems have previously been successfully used to improve air quality in human residential- and commercial buildings as well in agricultural settings. However, less is known about the efficacy of negative ionizing systems in commercial swine farrowing environments. Thus, the objective of this study was to use a swine farrowing environment to evaluate the effects of a negative air ionization system on 1) the quantity of airborne gaseous and particulate matter, and 2) swine health and production parameters. Six farrowing rooms containing 60 sows each were installed with 30 negative ionization systems per room. Three out of six rooms were randomly allocated between active ionization (L-ON) or inactive ionization (L-OFF) between farrowing rounds (N =  4). For each round, measurements of PM_2.5_, PM_10_, Ammonia (NH_3_), hydrogen sulfide gas (H_2_S), temperature, and humidity were collected twice a week, in the morning and afternoon at two heights, pig level (61 cm) and human level (152 cm). Pig performance metrics (parity, number of piglets born, number of live piglets born, piglet mortality, fostered piglets, and number of weaned pigs) were collected at the end of each batch. Comparisons between L-ON and L-OFF treatments were conducted by averaging room and day specific measurements for all days when rotating rooms shared contrasting treatments. Each room-specific L-ON treatment was then compared to all other L-OFF rooms using a linear regression model. No statistically significant differences were found between treatments for PM_2.5_ or PM_10_ at the pig nor human level. However, numerical reductions in the cumulative increase of PM_2.5_, and PM_10_ for L-ON rooms compared to L-OFF rooms were found in 60% of the L-ON rooms. One out of five L-ON rooms showed statistically slower buildup of NH_3_ concentrations compared to L-OFF rooms (*P* <  0.01) and 60% of the L-ON rooms had significantly slower buildup of H_2_S concentrations compared to L-OFF rooms (*P* < 0.01). No effect on production metrics were found between treatments. In conclusion, indications of improved air quality were found in this study, but given the complexity of these types of assessments, further work is needed to conclude the efficacy of negative ionization systems in commercial farrowing systems.

## Introduction

Suspended airborne particle matter (PM) pollution has been found to cause a range of health issues in humans such as, but not limited to, irritation of eyes and airways, increased inflammatory responses, asthma, arrhythmia, non-fatal heart attacks, vascular plaque formations, lung cancer, and triggered immune responses [1,2]. Suspended airborne particulate matter is generally defined as particle sizes that are not stopped by our nose and upper airways and therefore able to travel and be deposited deep into the lungs. These inhalable particles are classified into three category ranges which consists of coarse (2.5–10 μm, PM_10_), fine (1–2.5 μm, PM_2.5_) and ultrafine (<1 μm, PM_ < 1_) [3–5]. The new guidelines from the World Health Organization (WHO) state that the average daily exposure limit of PM_2.5_ and PM_10_ should not exceed 15.0 and 45.0 μg/m^3^, (or approximately 150 to 450 PPM/m^3^), respectively [6,7]. That said, average indoor PM_2.5_ levels have been reported to be around 7.3 to 25.0 PPM and can exceed well over 300.0 PPM during kitchen use or incense burning [8]. However, there is currently no consensus for maximum indoor PM concentrations for swine facilities which also vary depending on country, but a common threshold is set at 10 PPM [3,9,10]. That said, Donham et al. (2000) recommended a limit of 2.4 PPM for ≤ PM_10_ [9], while the Danish government has a threshold limit of PM of 3.0 PPM [3]. Additionally, exposure limits have been recommended to be set at significantly lower levels of 3.7 PPM and 0.23 PPM [9,11]. Particle matter pollution is an issue in commercial swine operations where air quality conditions can be poor due to a combination of poor ventilation, high animal density, dry animal feed and fecal matter [12]. Both short-term and long-term exposure to elevated levels of air suspended particulate matter in commercial swine farms can be a source of induced respiratory challenges in humans [9,13–16] and swine [5,17,18].

Air suspended particulate matter found in swine farms is mainly composed of a mix of organic matter from various sources within the farm such as the animal themselves, animal feed, fecal matter, and mineral dust and from outdoor sources such as pollen and mold [19,20]. The air suspension of particulate matter is highly dependent on relative humidity where high humidity levels increase particle agglomeration and deposition [21,22]. Moreover, air suspended particulate matter can chemically bond with gases such as ammonia (NH_3_), carbon monoxide (CO), carbon dioxide (CO_2_), methane (CH_4_) and hydrogen sulfide (H_2_S) regularly emitted into the air in commercial swine operations from urine and fecal matter as well as from the manure storage system [3,23,24]. Recent recommendations by the Italian Classyfarm guidelines for the maximum acceptable pollutant concentrations for indoor swine facilities have been suggested to be 0.5, 10, and 3,000 PPM for H_2_S, NH_3_, and CO_2_, respectively [3]. Human health risks linked from such gas emissions have been previously assessed both in- [25,26] and around swine facilities [27–29]. Out of the gases emitted from swine production facilities, H_2_S and NH_3_ are considered the most hazardous to both human and swine health. Prolonged and regular exposure to H_2_S and NH_3_ levels above 10 PPM and 25 PPM respectively may induce respiratory issues, sinusitis, chronic obstructive pulmonary conditions, and mucus membrane inflammation in humans [25,26] while in pigs, H_2_S levels above 20 PPM have resulted in photophobia, reduced or stopped feed intake and display of anxious behaviors [30]. Pigs may be more capable of coping with elevated NH_3_ levels compared to humans during certain circumstances. Previous studies have reported no effect on weight gain and feed intake as well as minimal to no gross pathology in weaner pigs exposed to NH_3_ levels up to 53 PPM for 5 weeks [31,32]. However, ammonia exposure around 10–15 PPM in piglets have been reported to cause issues such as turbinate atrophy, micro abscesses, and mucus membrane inflammation [33] and ammonia levels above 25 PPM may cause respiratory tract injuries, decreased immunity and growth performance and increased number of gram-negative bacteria in the nasal passageways [34].

The use of ionization systems has been successfully introduced to ameliorate poor air quality in indoor environments for both humans [35–38] and animals such as poultry [39–42], dairy [43] and swine [44,45]. Ionization systems mitigate poor air quality by producing negative ions (unipolar ionizers) or both negative and positive ions (bipolar ionizers) which attaches briefly to uncharged or ‘neutral’ particulate matter atoms and gases, causing them to deposit faster on nearby surfaces through static electrification, thus limiting the amount of air suspended particulates [46–48]. Recent advances in technology have allowed for the combination of ionization systems and commercial lighting fixtures, enabling a single solution for providing light and reduced air suspended particulate matter. However, evaluation of these options under commercial farm conditions is currently lacking in the literature.

Thus, the objective of this study was to evaluate the effects of a negative air ionization system on 1) the quantity of airborne gaseous and particulate matter, and 2) swine health and production parameters in a swine farrowing environment.

## Materials and methods

### Animals and study design

The study took place between June 2023 and Dec 2023 on a 5,000-sow commercial farrow-wean farm in the U.S. Midwest. The farm consisted of 16 farrowing rooms with 60 farrowing crates per room, and one large group housing gestation room. All study sows were housed in farrowing crates (244 ×  183 cm, length ×  width) on slatted metal (244 ×  61 cm) flooring with slatted plastic flooring in the creep areas (244 ×  61 cm). Manure was collected as slurry in underground pits and augured out according to farm standard operating protocols. Sows were housed mostly in an all-in all-out (AIAO) system as permitted by daily activities and adhered to standard commercial farm operation protocols and monitored daily for health problems or injuries. All caretakers had access to a treatment guide developed with the herd veterinarian, including common disease condition diagnosis descriptions and recommended treatment drugs and dosages. Sow information and production performance was obtained from electronic records in PigKnows (PigKnows LLC, Greeley, Colorado, USA).

**Fig 1:**
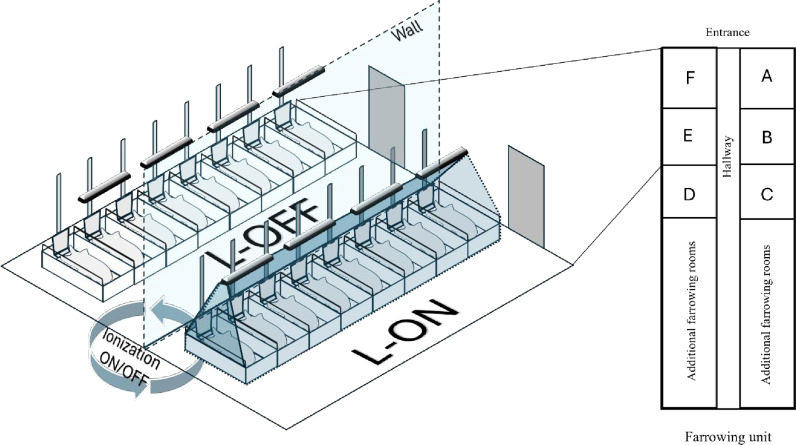
Example schematic of two farrowing rooms installed with (L-ON) or without (L-OFF) Freshlight^®^ ionization lights within the farrowing wing of a commercial swine site. Lights were installed in all rooms but the active ionization, indicated by a blue hue around the farrowing crates, was randomized between rooms and rotated between farrowing batches across farrowing rooms.

Six adjacent farrowing rooms (A-F) were used in this study. These rooms were selected due to ease of logistics (lights installation) and due to the fact that they were in close proximity to each other (Fig 1).

All farrowing rooms were fitted with 30 full spectra heavy duty Freshlight^®^ HDT 600 Series Nano tube lightning negative air ionization systems (NAIS) (Freshlight^®^ USA, Atlantic Beach, Florida, United States). The light fixture (128 ×  12.7 ×  10.2 cm, length, width, height) and LED light (62.5 ×  2.5 cm) had an ingress protection (IP)-67 rating capable for long-term use in a farm environment and installed at a height of 2.0 m spanning across one pair of farrowing crates. Each NAIS provided a single linear light source equal to 205 lumens per watt with a full light spectrum between 280 to 780 nm. Ionizers integrated into the lamp fixture had the capacity to produce up to 600 million negative ions per second capable of negatively charging airborne particles causing them to adhere to each other and other surfaces in the environment, reducing the number of suspended particles in the air. The six farrowing rooms containing the NAIS were divided into two groups: 1) Lights-ON (L-ON), where lights were kept on throughout the farrowing period and 2) Lights-OFF (L-OFF), where lights were hung but ionizers remained off. For both treatment groups, sow health and production data were collected across four independent farrowing batches, totaling 1,440 sows. Each farrowing room was power-washed and disinfected according to farm guidelines between each batch of farrowing sows.

### Particulate matter, gaseous measurements, and health and performance data

Particulate matter PM_2.5_ and PM_10_ were measured (μg/m^3^) by a handheld PM monitor (PCE-PCO 2, PCI-Instruments, PCE Americas INC, Jupiter, FL, USA). Hydrogen sulfide was measured in parts per million (PPM/m^3^) using a multi-gas detector (BW Clip 4, Honeywell International Inc, Charlotte, NS, USA) and ammonia was measured (PPM/m^3^) using an ammonia gas detector (BT-5800G, BTMETER, Zhuhai, China). PM and gaseous measurements were taken at a height of 61 cm and 152 cm twice a day (morning and afternoon) on two fixed weekdays (Monday and Friday) during each three-week farrowing batch. All measurements were taken at a predetermined location in the middle of the farrowing room by four trained caretakers on the farm; with measurements being instantaneous. The days of the week and times of daily measurements were selected to attempt for an average representation of activity levels throughout the week and days (beginning of the week immediately after the weekend/ beginning of day; and end of the week/end of day).

Treatment records containing sow- and piglet-ID, dates, health issue, and type of treatment were obtained from PigKnows. Sow-level performance data included parity, number of piglets born, number of live piglets born, piglet mortality, fostered piglets, and number of weaned pigs.

### Statistical analyses

A Spearman’s Rank correlation was performed to identify significant correlations between PM _2.5_, PM_10_, NH_3_, H_2_S, room temperature and humidity. Correlation coefficient (ρ) values of 0.1–0.3, 0.4–0.6, 0.7–0.9 or 1.0, were regarded as indicating weak, moderate, strong or perfect correlation, respectively [49].

Due to their physical location and layout within the barn, which determines exhaust fan locations, internal fan placements, number of access doors and general foot traffic, each room in our study had differences in ventilation and air flow, which influence gas and particle concentration over time. For the duration of our study, rooms had lights either on (L-ON) or off (L-OFF) each day. Using a fixed effects estimator, which compares the same room on different days, would be problematic because the air characteristics vary significantly from day to day due to the daily activities on a pig farm. Similarly, a random effects estimator was unsuitable as unobserved characteristics of the room may correlate with controlling variables such as temperature and humidity [50]. To account for this room-level variability, we developed linear regression models (one per PM/gas of interest) where measurements for each L-ON room were compared to a synthetic comparison room for the same day within the specific farrowing round. This synthetic comparison room was constructed by averaging the measurements of all L-OFF rooms on that day, allowing us to create a relevant benchmark that considers the environmental conditions specific to each day, providing a more robust comparison. This allowed for a day-to-day comparison of the effect of the L-ON on the degree of accumulative change per measured unit (μg/m^3^ for PM; PPM/m^3^ for NH_3_ and H_2_S) taking room and farrowing batch into account. As such, model coefficients > 1.0 indicate a measurement increase that exceeds baseline levels (L-OFF), while values < 1.0 indicate an increase slower than baseline levels. For instance, a coefficient of 0.9 μg/m^3^ for the synthetic comparison room should be interpreted as with every 1.0 μg/m^3^ increase in the synthetic room measurement there is 10% less increase in concentration buildup in L-ON compared to L-OFF rooms. One test room (F) had to be omitted due to insufficient comparative data for this specific model building due to chance from the randomization process of L-ON and L-OFF room allocation across farrowing batches.

Separate multivariable linear regression models were built to investigate the effect of humidity or temperature on PM_2.5_, PM_10_, NH_3_, H_2_S. For these models, humidity was dichotomized ( ≤ 64% or >  64%) based on previous studies showing increased PM_2.5_ and PM_10_ agglomeration and deposition at relative humidity levels above 64% [21,22]. Temperature was also dichotomized based on the mean room temperature during the study period ( ≤ 22.7% or >  22.7%). The variables treatment (L-ON, L-OFF), time (week 1–4), and time of day (morning, afternoon) were also included in this model as fixed effects; and Rooms (A-E) were nested within farrowing round and used as a random effect.

Finally, a linear regression model was built to investigate the effect of treatment on production parameters (number of piglets born, number of live piglets born, piglet mortality and number of weaned pigs) and health parameters (sow- and piglet-level treatments). To account for cumulative effects of air quality on production metrics, the maximum exposure was estimated based on the number of days each sow spent in the farrowing room and the room specific PM_2.5_-, PM_10_- NH_3_- and H_2_S-levels during the period. These cumulative maximum exposure variables were added as independent variables to the model together with temperature, humidity and parity. Room was nested within farrowing round to account for the rotation of treatments within rooms between farrowing batches.

All analyses were conducted using Stata 18 (College Station, Texas). Statistical significance within the final model was declared at *P < * 0.05, and tendencies were declared as 0.05 ≤  *P < * 0.10.

## Results

The Spearman Rank’s correlation analysis showed a strong positive correlation between PM_2.5_ and PM_10_ concentrations (ρ =  0.92, Table 1). In addition, a moderate negative correlation was observed for both PM_2.5_ and PM_10_ with relative humidity (ρ =  −0.44 and ρ =  −0.44, respectively). A moderate positive correlation was observed for NH_3_ with PM_2.5_ and PM_10_ (ρ =  0.53, and ρ =  0.57, respectively) and H_2_S (ρ =  0.55) but a negative correlation was found with relative humidity (ρ =  −0.45) (Table 1).

**Table 1:** Spearman Rank’s correlation matrix for temperature (Temp), humidity, PM_2.5_, PM_10_, H_2_S and NH_3_.

	Temp	Humidity	PM_2.5_	PM_10_	H_2_S	NH_3_
**Temp**	1					
**Humidity**	0.1903	1				
**PM**_**2.5**_	−0.4927	−0.4397	1			
**PM**_**10**_	−0.3511	−0.4367	0.9258	1		
**H**_**2**_ **S**	−0.1847	−0.1885	0.3566	0.3495	1	
**NH**_**3**_	−0.2073	−0.4461	0.5309	0.5724	0.5510	1

Descriptive mean (±SD) concentration levels of PM_2.5_ and PM_10_, humidity and temperature are presented in Table 2.

**Table 2:** Mean (±SD) particulate matter (PM) concentration (µg/m^3^) for PM_2.5_ and PM_10_ for farrowing rooms with (L-ON) or without (L-OFF) an activated ionizing Freshlight^®^ systems in farrowing rooms in a commercial swine facility during a 4-week farrowing cycle.

Variable	Category	PM_2.5_ (SD)	PM_10_ (SD)	Temp (C°)	Humidity (%)
Treatment	L-ON	521.5 (498.1)	856.4 (570.7)	22.8 (1.4)	69.4 (38.4)
	L-OFF	468.7 (313.0)	811.3 (292.8)	22.9 (2.0)	65.3 (10.2)
Week	1	267.6 (319.4)	657.2 (359.4)	24.0 (1.3)	74.9 (5.2)
	2	436.6 (321.1)	773.4 (316.4)	23.5 (1.5)	67.8 (9.4)
	3	620.2 (286.1)	916.1 (251.4)	22.2 (1.0)	64.5 (10.6)
	4	696.9 (604.7)	1034.5 (754.3)	21.7 (2.1)	60.6 (9.8)
Time	Morning	563.8 (466.5)	909.4 (509.7)	22.6 (1.8)	66.9 (9.1)
	Afternoon	425.3 (342.9)	757.3 (369.8)	23.3 (1.6)	67.7 (38.5)

At the pig-level (61 cm height), no significant differences for either PM_2.5_ nor PM_10_ were observed for day-to-day comparisons between L-ON and L-OFF rooms (Table 3). Numerical reductions in PM_2.5_ and PM_10_ concentration buildup were consistently observed for three L-ON rooms (B, C and E) while two rooms (A and D) displayed a numerically faster increase in PM_2.5_ and PM_10_ concentrations compared to other L-OFF rooms (Table 3). Numerical reductions in NH_3_ concentration buildup were observed for all rooms, with a statistical reduction in NH_3_ concentration buildup observed for L-ON room C (F_1,35_ =  8.09, *P* =  0.007) compared to L-OFF rooms. Four out of five rooms showed lower H_2_S concentration buildup, with statistically slower H_2_S concentration buildup observed for L-ON room C (F_1,36_ =  6.17, *P* = 0.02), D (F_1,22_ =  11.83, *P* =  0.002, and E (F_1,35_ =  7.75, *P* =  0.009) compared to L-OFF rooms.

**Table 3:** Air quality measurements for individual rooms (A-E) with active Freshlight^®^ ionizing lights for particulate matter (PM)_2.5_ and PM_10_, ammonia (NH_3_) and hydrogen sulfide gas (N_2_S) compared to rooms with inactive ionizing lights (baseline; L-OFF). Model coefficients > 1.0 indicate a measurement increase that exceeds baseline levels (L-OFF), while values < 1.0 indicate an increase slower than baseline levels.

Height	Measurement	Room A	Room B	Room C	Room D	Room E
*61 cm*	PM_10_^a^	1.396	0.948	0.722	2.094	0.912
	PM_2.5_^a^	1.159	0.813	0.751	2.115	0.836
	NH_3_^b^	0.781	0.868	0.525*	0.494	0.720
	H_2_S^b^	0.728	1.029	0.310*	0.057*	−0.027*
*152 cm*	PM_10_^a^	0.745	1.189	0.693	1.076	0.822
	PM_2.5_^a^	0.898	1.103	0.724	1.259	0.801
	NH_3_^b^	0.695	1.213	0.512*	0.824	0.802
	H_2_S^b^	0.982	0.925	0.136*	0.125*	0.116*

^a^Change per unit in μ/m^3^.

^b^Change per unit in parts per million (PPM)/m^3^_._

*Indicate statistical difference of *P* <  0.05.

At the human level (152 cm height), no significant differences for either PM_2.5_ nor PM_10_ were observed for day-to-day comparisons between L-ON and L-OFF rooms. Reduced concentration buildup was numerically observed for PM_2.5_ and PM_10_ for three L-ON rooms (B, C and E) while two rooms (A and D) displayed a numerically faster buildup in PM_2.5_ and PM_10_ concentrations compared to other L-OFF rooms (Table 3).

A significant reduction in NH_3_ concentration buildup was observed for L-ON room C (F_1,35_ =  9.14, *P* =  0.005) compared to L-OFF rooms (Table 3), with numerical reductions observed for three other rooms. Numerical reductions in H_2_S concentration were seen for all rooms, with a significantly slower buildup observed for L-ON rooms C (F_1,35_ =  9.21, *P* =  0.005), D (F_1,21_ =  6.36, *P* =  0.02) and E (F_1,35_ =  8.42, *P* =  0.01) compared to L-OFF rooms (Table 3).

**Fig 2:**
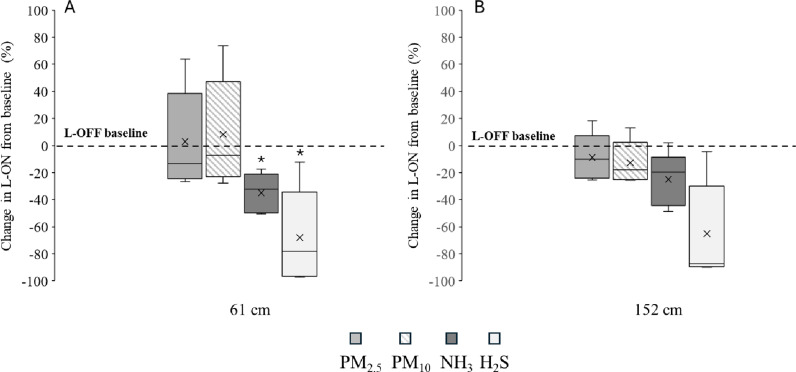
Change (%, ±  SE) in airborne PM_2.5_, PM_10_, NH_3,_ and H_2_S concentrations for L-ON rooms compared to baseline (L-OFF rooms) for 61 cm (A) and 152 cm (B). * Indicates significant difference of ***P*** <  0.01.

The overall changes (mean ±  SD) in PM concentrations for the L-ON rooms were numerically higher at 61 cm height with a 13.1% ( ± 57.3) and 21.1% ( ± 54.4) increase in PM levels throughout the study period for PM_2.5_- and PM_10_, respectively, compared to L-OFF rooms (Fig 2A). There was a significant reduction in NH_3_ and H_2_S concentrations built up at the 61 cm height level with a 32.2% (*P* =  0.01) and 57.0% (*P* =  0.04) slower increase compared to L-OFF rooms, respectively (Fig 2A). At 152 cm height, no significant treatment differences for PM_2.5_, PM_10_ or NH_3_ were observed, but a tendency for a slower buildup in H_2_S levels was observed in the L-ON rooms compared to L-OFF rooms (*P* =  0.06, Fig 2B).

No overall difference in humidity was observed between the rooms for each treatment (*P* =  0.10, Table 4). A significant week effect was observed across the farrowing period with decreasing humidity across weeks (*P* <  0.001, Table 4). Higher humidity levels were observed with increased temperature (*P* <  0.001, Table 4) and during the afternoon compared to the morning (*P* <  0.001, Table 4). No overall difference in temperature was observed between the rooms for each treatment (*P* =  0.45, Table 4). A significant week effect was observed across the farrowing period with decreasing temperatures over time (*P* <  0.001, Table 4). A room humidity above 64% resulted in higher room temperatures (*P* <  0.001, Table 4) and room temperatures were observed to be higher in the afternoon compared to the morning (*P* <  0.001, Table 4). No significant differences were observed regarding production metrics (Table 5).

**Table 4:** Results from mixed linear regression model for the effect of treatment (L-ON, L-OFF), week (1–4), time of day (morning, afternoon), humidity ( ≤ 64%, > 64%), and temperature ( ≤ 22.7, > 22.7 ^°^C) on humidity/ temperature in a commercial farrowing room.

Outcome	Variable	Category	Mean	SE	CI 95%	*P*-value
*Humidity*	Treatment	L-OFF (referent)	68.1	0.92	66.3–69.9	
		L-ON	65.9	0.92	64.1–67.8	0.10
	Week	1 (referent)	74.6	0.67	73.3–75.9	<0.001
		2	67.5	0.67	66.1–68.8	<0.001
		3	64.8	0.67	63.5–66.1	<0.001
		4	61.2	0.67	59.9–62.5	<0.001
	Time	Morning (referent)	66.6	0.66	65.3–67.9	
		Afternoon	67.5	0.67	66.1–68.8	0.006
	Temp	≤ 22.7 (referent)	66.9	0.66	65.3–67.9	
		> 22.7	67.2	0.66	66.1–68.8	<0.001
*Temperature*	Treatment	L-OFF (referent)	22.7	0.12	22.5–22.9	
		L-ON	22.9	0.12	22.7–23.2	0.17
	Week	1 (referent)	23.8	0.09	23.6–24.0	<0.001
		2	23.4	0.09	23.2–23.6	<0.001
		3	22.3	0.08	22.1–22.5	<0.001
		4	21.8	0.09	21.6–22.0	<0.001
	Time	Morning (referent)	22.4	0.09	22.2–22.6	
		Afternoon	23.3	0.09	23.1–23.4	<0.001
	Humidity	<64% (referent)	22.5	0.09	22.3–22.6	
		>64%	23.3	0.09	23.1–23.4	<0.001

**Table 5:** Descriptives (mean ±  SD) of production metrics per round and the number of sow and piglet health treatments for active (L-ON) or inactive (L-OFF) ionization system rooms.

Metric	Treatment	Round	Parity	Liveborn	Piglet Mortality	Weaned Piglets
*Production*	L-ON	1	3.1 ± 1.9	15.7 ± 3.3	1.6 ± 1.6	13.1 ± 1.6
		2	3.9 ± 2.2	15.2 ± 3.4	2.1 ± 2.1	12.3 ± 1.9
		3	3.4 ± 2.0	14.2 ± 4.2	2.1 ± 2.3	12.4 ± 1.8
		4	3.1 ± 1.6	14.8 ± 4.0	1.6 ± 1.9	12.9 ± 1.7
	L-OFF	1	3.3 ± 2.1	14.9 ± 3.2	1.9 ± 1.9	12.9 ± 1.8
		2	3.6 ± 2.4	14.8 ± 3.6	2.0 ± 2.1	12.5 ± 2.1
		3	3.6 ± 2.1	15.0 ± 3.9	1.9 ± 2.0	12.6 ± 1.8
		4	3.3 ± 2.0	14.6 ± 4.0	1.8 ± 1.9	12.7 ± 1.9
*Health*	**Treatment**	**Sow**	**Piglet**			
	L-ON	0.10 ± 0.55	0.28 ± 0.52			
	L-OFF	0.04 ± 0.29	0.26 ± 0.54			

## Discussion

To our knowledge, our study is the first to investigate the Freshlight^®^ HDT negative ionization system’s efficacy in reducing PM_2.5,_ PM_10,_ NH_3_ and H_2_S levels and improving health and production parameters in a commercial farrowing system. Within farrowing batches in such systems, it is expected that PM levels increase over time as animals stay within those rooms for longer periods of time and as cleaning procedures take place in between batches. Thus, as particles can’t be eliminated out of the system until the end of each farrowing batch, the main goal effect of the negative ionization technology would be to decrease airborne PM levels due to agglomeration and deposits rather than reduced PM levels.

This study showed numerically slower increases in PM levels between L-ON and L-OFF rooms throughout the study, where 3 out of 5 L-ON rooms showed a slower increase in PM_2.5_- and PM_10_-levels compared to L-OFF rooms. However, at the pig level (61 cm), the overall increase in PM concentration was faster by 13.1% and 21.1% in the L-ON rooms compared to L-OFF rooms for PM_2.5_- and PM_10_- respectively. This effect was mainly driven by two rooms which showed a 30% and 100% higher increase in PM concentrations compared to L-OFF rooms. In contrast, at the human level (152 cm), there was a slower increase of 3.9% and 8.4% in the L-ON compared to L-OFF rooms for PM_2.5_- and PM_10_- respectively.

A previous pilot study using the same Freshlight^®^ HDT negative ionization system found a reduction in PM_10_ levels by 41.0% in a commercial cage-free layer barn [51]. Other systems such as electrostatic particle ionization (EPI) have been investigated in pig environments and shown a 40–60% reduction in air suspended PM_0.3_-PM_10_ in the weaning pig environment [52] and a Bionaire negative ionization system resulted in a 28–46% reduction for PM < 5.0 μm [39]. Moreover, the use of an EPI system in broiler houses reported PM_10_ and PM_2.5_ reductions of 36% and 10%, respectively [53], while vastly different results have been reported using electrostatic plate precipitator systems (ESP) with reductions varying from around 6.5% and 6.7% [54] to 57% and 45.3% [55] for PM_10_ and PM_2.5_, respectively. Additionally, a study using negative air ionization in broilers and positive ionization systems in layers found a 49% and 68% reduction in broilers and a 6% and 0% reduction in layers for PM_10_ and PM_2.5_, respectively [55]. Thus, there is some evidence that the efficacy of different ionization systems may be environment specific as previous studies have shown varying degrees of PM reductions. It is therefore possible that, although the system examined herein showed significant reductions in a layer environment, the efficiency in a farrowing environment may be different or require a different approach for full functionality. That said, we did see numerical reductions of PM_2.5_ and PM_10_ in our study at the human level (152 cm) which could be interpreted as that the system may be effective in reducing the PM load higher up in the air column, but the effect of increasing PM levels over 4 weeks may be deemed challenging in a relatively enclosed space as PM can be re-suspended due to animal- and human activity. A secondary issue contributing to increased PM levels over time is the dry feeding system used in the farrowing rooms. This type of feeding system contributes to PM levels as feed is delivered using an auger followed by a gravity induced drop into the feed hopper which would resuspend PM into the air column [56,57]. Similarly, numerical reductions in NH_3_ and H_2_S were observed in 9 out of 10 L-ON rooms compared to L-OFF rooms. This is in line with findings that did not see any significant reductions in NH_3_ and H_2_S using a EPI system for a swine weaning environment [52].

Humidity and temperature may also play a part in the efficacy of the system. It is known that high humidity increases particle agglomeration and deposition, especially when humidity levels exceed 64% [21,22]. Although humidity levels in the L-OFF treatment were numerically higher compared to L-ON rooms, no statistical difference was found. In addition, the average humidity levels were well above 64%, making it hard to explain the lack of difference between treatments due to humidity agglomeration more than the ionization process. In fact, additional humidity has shown to increase electrostatic forces in PM, thus accelerating agglomeration and therefore the effectiveness of the ionization process in our L-ON treatment [58]. Additionally, as rooms were allocated on a rotational schedule between farrowing rounds, and the humidity remained similar between L-ON and L-OFF rooms, any potential room bias throughout the study period should have been mitigated.

We did not see any differences in production metrics between the L-ON and L-OFF treatments. Regardless of the non-significant PM reductions in our study, it is likely that air quality improvements during a relatively short time span of the pigs’ life don’t have enough time to manifest itself in productivity. However, production issues in swine have previously been linked to poor air quality [59,60]. Future large-scale studies should consider following individual pigs throughout multiple farrowing rounds to assess the long-term impact of Freshlight^®^ HDT ionization lights.

Anecdotally, there may also have been unmeasured positive side-effects of introducing the ionizing lights in the farrowing system. According to the caretakers on the site, they perceived the luminance of the Freshlights^®^ to be higher compared to the lights traditionally used, which helped cleaning procedures in between farrowing rounds. In addition, some of the caretakers perceived the air quality to be better in the test rooms during the trial period compared to other farrowing rooms. Improved cleaning between farrowing rounds due to improved lighting could have a positive effect on base level cleanliness and reduced pathogen loads and should be investigated further.

Our study does not come without limitations. First, only two weekly measurements were taken throughout the study period, and considerable variation in particle concentrations were observed throughout the study, which made it difficult to control for. The use of synthetic controls should have helped with this issue by ‘normalizing’ the data by day. Furthermore, even though many sows and piglets were included during the four examined batches, treatment allocation needed to be at the room level, which limited sample size during the analytical stages. It is also important to note that only six rooms within one commercial sow farm were used; and as such results may not be generalizable for farms of other swine age ranges. Future studies should focus on utilizing this technology for longer durations of time (e.g., across different seasons), as well as on a larger number of sites, using continuous aerosol particle monitoring systems and monitoring for finer PM. We also acknowledge that the commercially available sensors utilized to measure particles were not tested for measurement accuracy in our study. However, if there were measurement errors in the equipment, that would likely have been applied consistently across treatment groups, decreasing its potential to bias the results. Finally, adding surveys of swine caretaker perceptions of cleaning procedures, cleanliness and air quality before and after Freshlight^®^ installation to future studies are encouraged.

## Conclusions

This study is the first to investigate the efficacy of a Freshlight^®^ HDT ionization system on PM_2.5_, PM_10_, NH_3_ and H_2_S concentrations in a commercial swine farrowing system. Although no significant differences between rooms with or without the ionization system was found, trends of air quality improvements were seen, especially for NH_3_ and H_2_S where individual farrowing rooms showed significant decreases in concentration buildup during a 4-week period. The efficacy of air ionization systems is highly situation dependent, and additional review and investigation of Freshlight^®^ light fixture density and placement height is needed. Finally, implementation of continuous air monitoring would help establish better data to account for PM variability over time.

## Supporting information

S1 Data Minimal data. (XLS)
